# Cardiac autotransplantation and ex vivo surgical repair of giant left atrium: a case presentation

**DOI:** 10.1186/s12872-018-0966-2

**Published:** 2018-12-18

**Authors:** Zan Mitrev, Milka Klincheva, Tanja Anguseva, Igor Zdravkovski, Rodney Alexander Rosalia

**Affiliations:** Zan Mitrev Clinic, Bledski Dogovor 8, Skopje, 1000 Republic of Macedonia

**Keywords:** Giant left atrium, Warm blood perfusion, Atrial fibrillation, Cardiac autotransplantation

## Abstract

**Background:**

Chronic Mitral Valve disease is strongly associated with Left atrial enlargement; the condition has a high mortality risk. Clinical manifestations include atrial fibrillation, pulmonary hypertension, thromboembolic events, and in cases of Giant Left Atrium (GLA) and a distorted cardiac silhouette. Full sternotomy, conventional open-heart surgery, reductive atrioplasty and atrioventricular valve repair are required to resolve symptoms. However, these procedures can be complicated due to the posterior location of the GLA and concomitant right lateral protrusion.

Cardiac autotransplantation is superior under these conditions; it provides improved visual access to the posterior atrial wall and mitral valve, hence, facilitates corrective surgical procedures.

We aimed to assess the clinical outcome of patients undergoing cardiac autotransplantation as the primary treatment modality to resolve GLA. Moreover, we evaluated the procedural safety profile and technical feasibility.

**Case presentation:**

Four patients, mean EuroSCORE II of 23.7% ± 7.7%, presented with heart failure, atrial fibrillation, left atrial diameter > 6.5 cm and a severe distorted cardiac silhouette; X-ray showed prominent right lateral protrusion.

We performed cardiac autotransplantation using continuous retrograde perfusion with warm blood supplemented with glucose followed by atrioplasty, atrial plication, valve annuloplasty and valve repair on the explanted beating heart. The surgical approach reduced the left atrial area, mean reduction was − 90.71 cm^2^ [CI95% -153.3 cm^2^ to − 28.8 cm^2^, *p = 0.02*], and normalized pulmonary arterial pressure, mean decrease − 11.25 mmHg [CI95% -15.23 mmHg to − 7.272 mmHg, *p = 0.003*]. 3 out of 4 patients experienced an uneventful postoperative course; 2 out of 4 patients experienced a transient return to sinus rhythm following surgery. One was operated on in 2017 and is still in good condition; two other patients survived for more than 10 years; Kaplan-Meier determined median survival is 10.5 years.

**Conclusions:**

Cardiac autotransplantation is an elegant surgical procedure that facilitates the surgical remodelling of Giant Left Atrium. Surgical repair on the ex vivo beating heart, under continuous warm blood perfusion, is a safe procedure applicable also to high-risk patients.

## Background

Chronic mitral valve diseases are considered the primary trigger for pathological Left Atrial Enlargement (LAE) [1–3]. LAE is predictive for adverse outcomes and is independently related to cardiovascular morbidity and mortality [4, 5].

Untreated chronic mitral valve disease predisposes the patients to secondary illnesses such as pulmonary hypertension [6, 7], respiratory complications [8] thromboembolic events [9] and atrial fibrillation (AF); the degree of the left atrium morphological and functional abnormalities determine the severity of AF [10–12].

In rare cases, with an estimated 0.3–0.6% incidence rate, patients present with Giant Left Atrium (GLA), characterised by an LA diameter > 6.5 cm or an LA area >  40 cm^2^ [2, 5, 13]. Patients with GLA frequently present with a severely distorted cardiac silhouette and abnormal cardiothoracic ratio. LA protrusion into the right chest cavity may be observed via X-ray. Moreover, these patients often suffer from chronic AF [12]. Circumferential corrective surgery of GLA would ideally require complete cardiac explantation, ex vivo surgical remodelling and autotransplantation.

Cardiac autotransplantation is mostly reserved to debulk cardiac tumours [14–16]. However, it offers distinct advantages to achieve efficient surgical remodelling; cardiac autotransplantation is highly suitable for reductive (left) atrioplasty as the ex vivo handling of the heart allows full access to the posterior left atrial wall and complete visualisation of the mitral valve.

This paper describes our experience with cardiac autotransplantation, and concomitant cardiac corrections, in the treatment of four patients who presented with cardiac and pulmonary complications because of chronic mitral valve disease. All patients presented with NYHA IV and GLA.

Our observations point to a substantial clinical benefit, emphasised by a decrease in symptom severity, successful cardiac remodelling, restoration of left atrial morphology, normalisation of pulmonary hypertension and a median survival time of 10.5 years in the absence of postoperative complications.

### Surgical technique

Patients (Table 1) were operated using general cardiac anaesthesia. We access the heart via a median sternotomy. Surgery was performed under cardiopulmonary bypass (CPB), without cardiac arrest under normothermic conditions (> 34 °C) applying bicaval venous cannulation, coronary sinus (CS) cannulation and continuous retrograde warm blood perfusion - beating heart methodology. We controlled the mean systemic arterial pressure at 65 mmHg and used a blood auto-reinfusion system (auto trans®) in all cases.

**Table 1:** Patient characteristics, diagnosis, echocardiography parameters and overview of surgical procedures

Patient/Case #	1	2	3	4
Age	42	64	65	64
Gender	M	M	F	F
Date of Hospitalisation	29/11/2001	30/03/2003	15/03/2004	28/09/2017
Weight (Kg)	48	61	50	63
Height (m)	1.86	1.58	1.49	1.6
BMI (kg/m2)	13.9	24.4	22.5	24.6
BSA (m2)	1.57	1.64	1.44	1.67
EuroSCORE II (%)	16.4	18.3	28.9	31.9
Aetiology	Congenital	Degenerative	Rheumatic	Degenerative
Performed cardiac corrections & Surgical Remodelling				
Reductive Left Atrioplasty	Yes	Yes	Yes	Yes
Reductive Right Atrioplasty	No	Yes	No	Yes
Mitral Annuloplasty	Yes	Yes	No	No
Mitral Valve Replacement	No	No	Yes	Yes
Tricuspid annuloplasty	Yes	Yes	No	Yes

*BMI* Body mass index

*BSA* Body Surface Index

*EuroSCORE II* European System for Cardiac Operative Risk Evaluation (II)

Symbols adjacent to the patient case # correspond to the symbols used in Fig. 2 to facilitate individual analysis

**Fig. 1:**
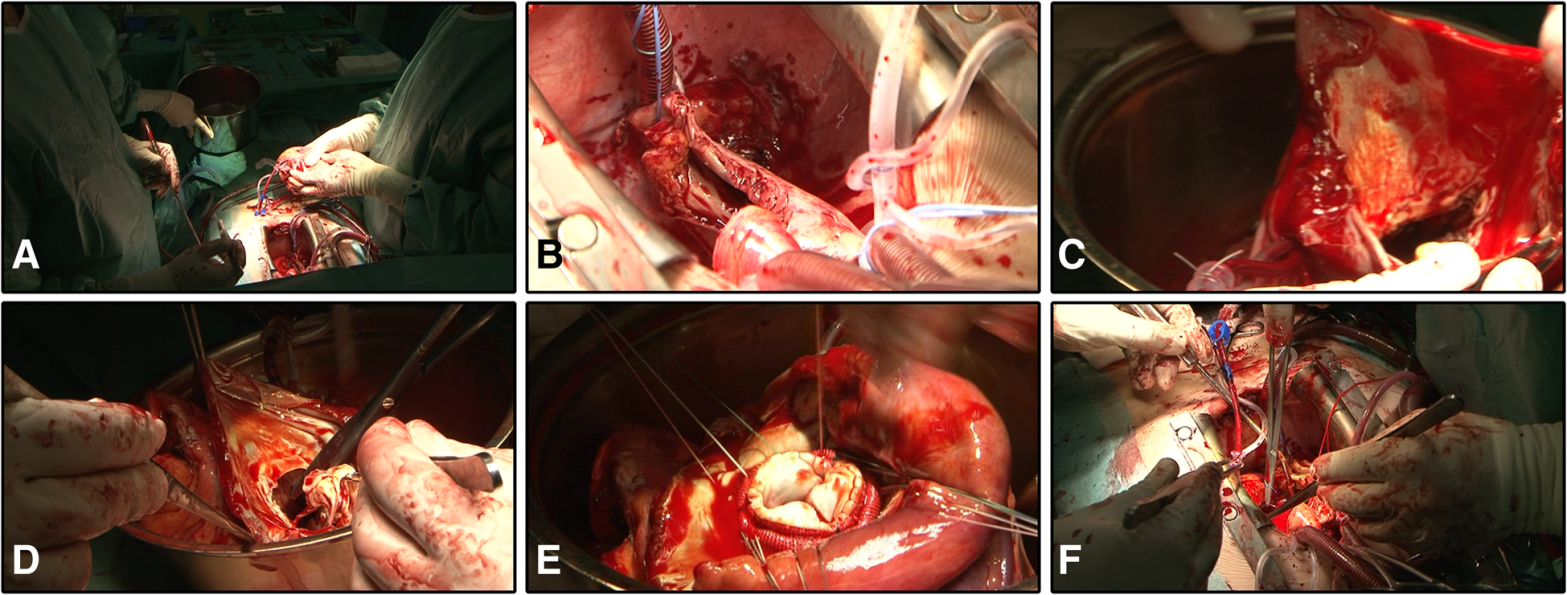
Intraoperative images of the cardiac autotransplantation procedure. **a** Explanted and ex vivo handling of the enlarged heart. **b** Preserved, cannulated vena cava and residual Right Atrial wall tissue. **c** The open left Atria with the diseased mitral valve. **d** The full excision of the diseased mitral valve. **e** Implantation biological valve prosthesis. **f** depicts the sternum with the re-implanted, surgically corrected heart

Cardiac autotransplantation (Fig. 1) is initiated via a bicaval cannulation of the superior and inferior vena cava. A catheter is placed in the CS ostium using a purse-string suture for retrograde perfusion; we continuously perfuse the heart with warm blood supplemented with 50% Glucose – flow rate is maintained at 200 ml/min, and the in-vessel-pressure set at 40–50 mmHg.

Next, the aorta is cross-clamped followed by the transverse cut of the aorta and the pulmonary trunk. At this stage, we perform a longitudinal cut in the posterior wall of the LA. We preserve the vena cava and perform the incision in the interatrial septum followed by a cut along the crista terminalis of the right atrium (RA). In summary, our cardiac autotransplantation method allows the explantation of the cannulated heart while preserving the major veins.

Surgical cardiac remodelling was performed combining atrial reconstruction, reductive atrioplasty - rough debulking of excess atrial tissue - and left atrial appendage excision and concomitant Cox-Maze procedure [17]. Following surgical remodelling and autotransplantation of the heart, we reconnect the LA to its base, the anastomosis of the aorta and pulmonary artery are performed, and the RA sutured and closed. The aorta, pulmonary artery and both caval veins are unclamped and weaning from CPB is initiated.

Table 2 describes the total surgery duration, cardiopulmonary bypass time, aortic cross-clamp time and duration of perioperative respiratory support.

**Table 2:** Preoperative, Intraoperative and postoperative patient characteristics

Patient/Case #	1	2	3	4
Preoperative data				
MV regurgitation (grade)	Severe	Severe	No	Severe
MV Stenosis (grade)	No	No	Severe	No
MV annulus (cm)	5.5	4.4	4.0	4.6
TV regurgitation (grade)	Severe	Severe	Moderate	Moderate
TV annulus (cm)	4.1	3.8	2.7	3.7
LA diameter (cm) (Fig. 2)	13	17	9.2	9
LA area (cm^2^)	143	221	120	81
IVSd (cm)	12	12	10	11
LVPWD(cm)	12	12	10	11
LVEDd (cm)	6.8	6.0	6.2	5.1
LVEDs (cm)	3.5	3.5	3.8	3.4
LVEF (%) (Fig. 2)	40	40	35	45
SPAP (mmHg) (Fig. 2)	> 40	> 40	> 35	> 30
NYHA classification	IV	IV	IV	IV
Coronary angiography	Normal	Normal	Normal	normal
Perioperative data				
Cardiopulmonary bypass (min)	87	135	253	135
Aorta cross-clamp time (min)	48	73	177	82
Total operation duration (min)	215	250	338	195
Mechanical ventilation (hours)	96	46	96	24
ICU LOS (hours)	202	3	7	14
Total Hospitalisation (days)	202	19	15	20
Postoperative analysis				
MV annulus (cm)	3.2	3.0	3.1	2.7
MV regurgitation (grade)	No	No	No	No
MV stenosis (grade)	No	No	No	No
TV (grade)	No	No	No	mild
LA diameter (cm)	6.5	5.6	3.5	4.7
LA area (cm^2^) (Fig. 2)	42.5	36	16.8	29.8
LVEF (%) (Fig. 2)	50	40	40	50
Complications	Fever	No	Mild fever	No
SPAP (mmHg) (Fig. 2)	30	30	20	20
NYHA classificaton	–	II	I	I
Follow up (days)	214	3816	4855	249
Postoperative Sinus rhythm duration (days)	0	364	0	5
Intrahospital mortality (days)	214	No	No	No
Cause of death	Mesenteric ischemia	Sepsis	Malignancy	–

*MV* Mitral Valve

*TV* Tricuspid Valve

*LA* Left Atrium

*IVSd* Interventricular Septal Thickness at Diastole

*LVPWD* Left ventricular posterior wall dimension

*LVEDd* Left ventricular diastolic diameter

*LVESd* Left ventricular systolic diameter

*LVEF* Left Ventricular Ejection Fraction

*SPAP* Systolic Pulmonary Arterial Pressure

*NYHA* New York Heart Association

SPAP is measured via a *Swan*-*Ganz catheter*

**Fig. 2:**
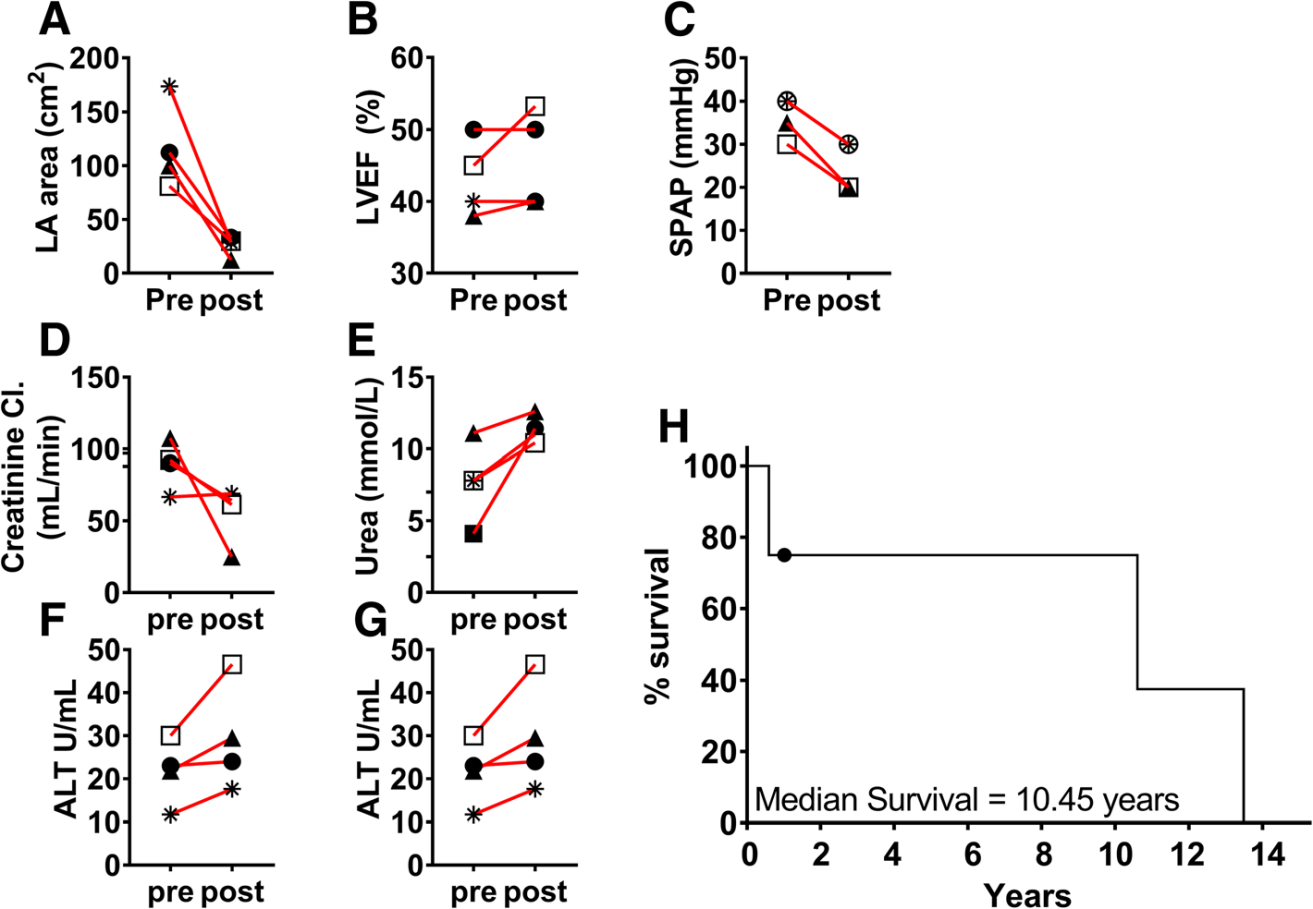
Perioperative Echocardiography, Laboratory analysis results and Survival. Panels depict the paired pre- and postoperative measurements for Left Atrium area (**a**), Left Ventricular Ejection Fraction (**b**) and Pulmonary Arterial Pressure (**c**). Panels show the paired pre- and postoperative creatine clearance (**d**), blood Urea levels (**e**), the liver enzyme Alanine transaminase (**f**) and Aspartate transaminase (**g**) levels. The estimated median survival following cardiac autotransplantation to perform atrial corrective surgery and atrioventricular valve reconstruction or replacement, censored subject (patient #4) is indicated, (**h**). Symbols in graphs correspond to the patient case # described in Tables 1 and 2

Symbols adjacent to the patient case # correspond to the symbols used in Fig. 2 to facilitate individual analysisv

### Case series summary

Four patients with left atrial cardiomegaly and concomitant atrial fibrillation were treated at our clinic in 2001, 2003, 2004 and 2017. Table 1. describes the basic patient characteristics and the corrective surgical procedures and Table 2. presents a summary of the perioperative echocardiography examinations and duration of care.

Primary symptoms reported were dyspnoea and angina. All patients presented with NYHA class IV. Three patients presented with syncope; we observed peripheral oedema in 2 patients. Echocardiography showed a reduced left ventricular contractility, LVEF 43.3 ± 5.4%, with no distinct outliers among patients. All patients presented with severe mitral valve disease, we observed 3 cases of grade 4 regurgitation and one case of severe MV stenosis.

Surgical corrections performed were atrioplasty, valve annuloplasty or replacement (Table 1). Three out of four patients experienced a swift and uneventful recovery. The procedure successfully reduced left atrial area, mean difference 90.71 cm^2^ [CI95% 153.3 cm^2^ to 28.8 cm^2^], *p = 0.02* (Fig. 2a), stabilised LVEF (Fig. 2b) and reduced the pulmonary hypertension by (mean) 11.25 mmHg [CI95% 15.23 mmHg to 7.272 mmHg], *p = 0.003* (Fig. 2c).

In these three patients, we could control the minor decrease in renal and liver function without artificial support (Fig. 2d–g). Two out of four patients experienced transient relief from AF, of which one patient converted to and remained in sinus rhythm for nearly 1 year after surgery (Table 2). The median survival was 10.5 years in the absence of MACCE (Fig. 2h).

Long-term follow up confirms a sustained atrial reverse remodelling after 10 and 13 years, respectively.

#### Patient 1

A 42-year-old male patient with a 21-year cardiac medical history presented at our emergency department in 2001. His symptoms worsened in the days preceding the surgical intervention. Upon examination, he was heavily dyspneic, with severe palpitations, worsening chest discomfort, coughing and haemoptysis.

Since 1983, on numerous occasions he was advised to undergo cardiac surgery to alleviate his symptoms; however, the patient was unable to decide on surgery.

Transthoracic echocardiography (TTE) revealed a Giant Left Atrium (GLA), dilated cardiomyopathy, prominent right atrial protrusion, and hemodynamically significant mitral and tricuspid regurgitation (Table 2). Computed tomography showed mid-oesophageal and bilateral pulmonary compression from the left atrium combined with congenital bilateral bullous emphysema – specific for congenital lung cystic emphysema. Cardiac autotransplantation and surgical remodelling were successful (Table 1).

Nevertheless, the patient experienced several respiratory complications related to the underlying congenital disease. Also, he experienced thrombocytopenia and excessive bleeding during the postoperative course.

Severe bacterial pneumonia and recurrent pneumothorax further complicated the clinical condition. The patient required prolonged ventilation support and surgical tracheostomy.

His condition further deteriorated in the following months. Our examinations revealed severe mesenteric ischemia with the involvement of the ileum at day 190; This complication was finale fatal after 202 days.

#### Patient 2

We hospitalised a 65-years-old man, in 2003, complaining of fatigue, dyspnoea, heart palpitations, hepatomegaly and peripheral oedema. TTE revealed severe mitral and tricuspid regurgitation resulting from myxomatous degeneration of both valves, with significant atrial cardiomegaly (Table 2).

The postoperative course was uneventful. Due to progressive rheumatic disease, 4 years after the surgical procedure the patient developed high-grade atrioventricular block for which a single chamber pacemaker was implanted; no other cardiac abnormalities were detected since then.

The patient succumbed to an acute septic shock, as a consequence of a neglected right lateral incarcerated inguinoscrotal hernia, after 10 years and 5 months.

#### Patient 3

A 65-years-old woman was referred to our hospital, in 2004, with severe mitral valve stenosis that manifested with severe chest pain, fatigue, dyspnoea, hepatomegaly and peripheral oedema. Her symptoms started 8 years before her hospitalisation.

She had an episode of rheumatic fever when she was 10 years old and had undergone a left nephrectomy.

TTE indicated severe mitral stenosis with severely enlarged left atrium (Table 2). Mitral valve replacement, reconstruction of the tricuspid valve and surgical remodelling of the left atrium was performed on the explanted heart. The patient recovered swiftly in the absence of postoperative complications.

The patient experienced a symptom-free postoperative course until 2016 when she complained about significant chest pain. Magnetic resonance imaging detected bone metastasis; the patient refused further medical care and passed away, presumably from cancer, mid-2017.

#### Patient 4

**Fig. 3:**
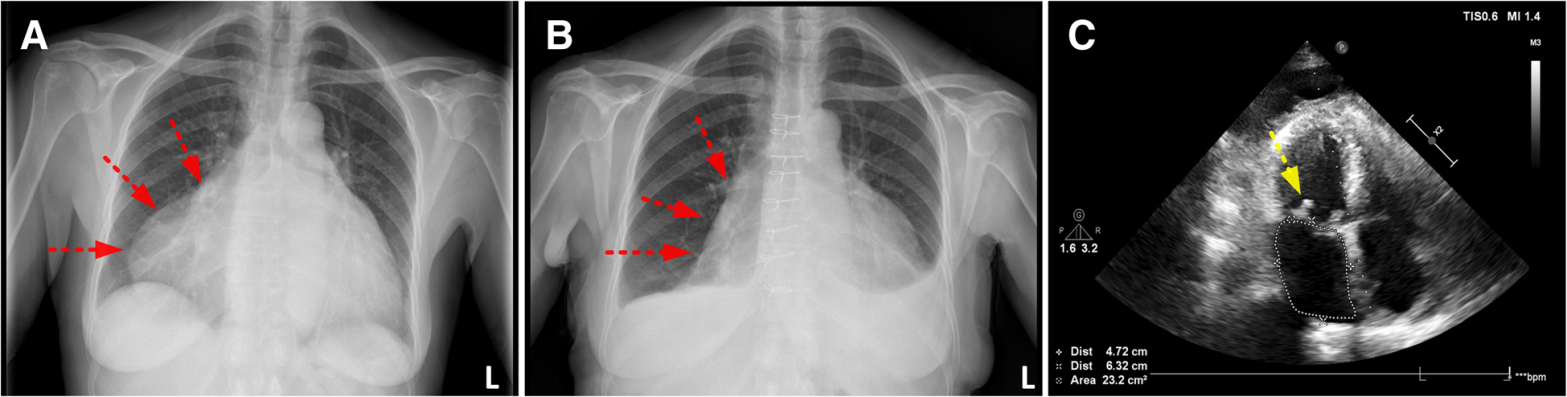
The Radiological examinations of a patient with GLA and right lateral protrusion. Pre- (**a**) and postoperative (**b**) AP X-ray images of the cardiac silhouette of a 65-year-old woman with severe mitral regurgitation, pulmonary hypertension, tricuspid regurgitation and dilation of the left atrium (LA area, 81 cm^2^ and a cardiothoracic ratio of 0.8). Red arrows point to the right heart border (**a** and **b**). Heart auto-transplantation was performed to replace the mitral valve with a biological prosthesis (yellow arrow) (St. Jude Medical, 27 mm). Excess atrial tissue was surgically removed followed by LA atrioplasty. Red arrows point to the right-lateral lining of the cardiac silhouette (**a** and **b**). The procedure successfully restored normal concave left heart border (**b**), (LA area, 23 cm^2^ and a cardiothoracic ratio of 0.6). Postoperative echocardiogram displayed a normalised left atrium area (indicated by the dotted line) and other chambers without significant morphological distortions (**c**)

We hospitalised a 64-year-old woman due to palpitations, fatigue, dyspnoea, and giddiness. TTE revealed severe mitral and tricuspid regurgitation (Table 2) - due to myxomatous degeneration - with bi-atrial enlargement. X-ray analysis indicated a significantly enlarged left atrium. Subsequent confirmed the case of GLA, indicated by a distorted cardiac silhouette and a cardiothoracic ratio of 0.8 (Fig. 2a). Given the high degree of right lateral protrusion and LA dimensions, we opted for cardiac autotransplantation (Fig. 1) in order to perform mitral valve replacement, tricuspid valvuloplasty and reductive atrioplasty. The Intrahospital postoperative course was uneventful. Several check-ups at our outpatient clinic during the first postoperative year confirmed her improved clinical condition, improving cardiac silhouette, a better cardiothoracic ratio of 0.6 (Fig. 3) and a normalised left atrial area of 23 cm^2^ (Fig. 2a). We noted only low-grade residual mitral insufficiency and AF that is successfully managed using anti-coagulation medication and conventional medical treatment.

## Discussion and conclusions

Mitral Valve diseases are associated with high morbidity and mortality [1–3]. Mitral valve dysfunction, but also other congenital disorders such as Atrioventricular (AV) Septal Defect (AVSD), result in an increased intra-atrial pressure leading to constant strain and dilation of the left atrial chamber. Consequently, these diseases trigger progressive pathophysiological changes to the left atrial structure and mechanical function.

Chronic disease can lead to severe LA dilatation; Giant left atrium (GLA), characterised by a left atrial diameter exceeding 6.5 cm [5]. Left atrial dilatation is strongly associated with the onset of AF [11].

Oral anticoagulants and minimally invasive ablation techniques have improved the care of AF patients; these treatments can directly or indirectly trigger LA reverse remodelling and thus normalise left atrium dimensions [18–23]. Nevertheless, in some instances, treatment failure results in persistent pathogenic LA remodelling and consequently, GLA.

Corrective surgery is indicated as the primary approach to resolving severe mitral valve dysfunction and GLA [5, 24, 25].

Several surgical techniques have been described for the treatment of GLA. Conventional cardiac surgery [5], partial autotransplantation [26–28] and orthotopic heart transplantation [29] have been performed to resolve GLA and mitral valve disease with varying success [5].

Complete cardiac explantation was initially devised to excise malignant tissues posterior located in the left heart [30, 31], difficult to reach through standard open-heart surgery. Caution is warranted because of the high morbidity and mortality rates associated with the procedure; especially when GLA corrective surgery is accompanied by mitral valve corrections [32–34]. Consequently, not many surgeons attempt cardiac autotransplantation.

One successful case of cardiac autotransplantation was previously reported [29] with a 30-day follow up; few studies have evaluated cardiac autotransplantation as a treatment modality for mitral valve diseases with concomitant GLA.

Nevertheless, we show that cardiac autotransplantation is reasonably safe and offers substantial clinical benefits – it allows efficient cardiac surgical remodelling and resolves symptom severity. 3 out of 4 patients recovered well postoperatively and experienced a generally good quality of life. One patient suffered a postoperative aggravation related to his congenital lung disease which proved fatal. Despite the unfortunate outcome, the surgery was technically successful and did relief the patient from his cardiac complications and related clinical symptoms.

As mentioned previously, left atrial dilatation is a risk factor for AF [11, 35]. Surgical management, reductive atrioplasty and concomitant Cox maze procedures [17] have been shown to efficiently restore sinus rhythm in a select patient population [5, 36]. For example, attaining postoperative normal atrial size was critical in restoring sinus rhythm [5]. Despite several negative predictive factors, namely long-term episodes of AF, two out of four patients experienced a transient return to sinus rhythm. Although all patients relapsed, we could effectively manage AF through medication and regular clinical examinations.

Our cardiac autotransplantation technique mirrors the methods initially described by Cooley et al.,1985 [31] and, more recently, by Reardon et al., 2010 [37]. We adapted these elegant surgical methods by 1) preserving both caval veins and performing the cut along the crista terminalis of the RA, and 2) by using continuous retrograde perfusion using warm blood supplemented with 50% Glucose, and 3) completing all cardiac corrections on the beating heart.

At our clinic, most conventional open-heart valve repair/replacement surgeries and CABG procedures are performed on the beating heart; our approach to cardiac protection, *on-pump/continuous perfusion of the beating heart with warm blood supplemented with glucose*, leads to shorter reperfusion times after aortic unclamping and faster postoperative recovery.

A technical limitation of our study is the use of 2D-measurements to diagnose and follow-up our patients. Volumetric approaches offer higher sensitivity to asses LA abnormalities than LA diameter-based measurements. Nonetheless, the four patients described here presented with such severe GLA that both analytical methods would suffice to diagnose their condition. Furthermore, the X-ray and CT examinations complemented our 2D-echocardiography measurements. To this end, our multi-modal diagnostic approach successfully identified GLA and allowed us to set up a successful surgical treatment modality based on cardiac autotransplantation [11, 38].

We are particularly interested in determining whether our surgical corrections promote long-term post-operative structural and functional atrial reverse remodelling [39]. The longitudinal evaluation of the LA area revealed a stable diameter. (patient #1 was excluded from the longitudinal analysis due to Intrahospital death). In patients #2 and #3, the LA area remained stable as assessed at 10.5 and 13.4 years, respectively. Interestingly, in patient 4, our most recent case, a further reduction in the LA area to 15.2 cm^2^ was observed after 172 days, from 29.8 cm^2^ Long-term follow up of this patient will offer insights to the continuity of the left atrial reverse remodelling. To the best of our knowledge, this case series, albeit based on a small cohort, for the first time describes the long-term clinical outcome of patients who underwent cardiac autotransplantation.

It is plausible that the continuous perfusion of the explanted beating heart with glucose-supplemented warm blood, during the corrective surgical procedures, helps cardiac reverse remodelling by supporting the foetal gene and metabolic programs of the damaged myocardium, hence promote recovery [40, 41].

The clinical management of GLA requires an individual approach tailored to the specific cardiac abnormalities. Few cases on the successful treatment of GLA have been reported in the literature with limited follow-up period. Data sharing among other clinical centres and evaluation of new patients might identify an optimal method to treat GLA; nevertheless, based on the long-term assessment of the patients described here, cardiac autotransplantation seems promising.

In conclusion, cardiac auto-transplantation is a safe approach to address GLA and underlying atrioventricular valve diseases surgically. Size reduction proved to be stable in the long run.

## Abbreviations

AF: Atrial Fibrillation
GLA: Giant Left Heart
LA: Left Atrium
LV: Left Ventricle
RA: Right Atrium
SVC: Superior Vena Cava
